# A Preliminary Satisfaction Study on Older Adults Dementia Knowledge Using a Game‐Based Learning Platform

**DOI:** 10.1002/alz70860_106585

**Published:** 2025-12-23

**Authors:** Angie L Sardina, Brynn Market, Lauren Desing, Betsabe Diaz, Darius Fullenwinder, Jeffrey Meadows, Marsha Hampton, Lena Simon, Lesley A. Ross, Alyssa A. Gamaldo

**Affiliations:** ^1^ Clemson University, Institute for Engaged Aging, Seneca, SC, USA

## Abstract

**Background:**

Game‐like digital technologies are beneficial for older adults as they facilitate learning and enrich the quality of aging through knowledge and empowerment. While various studies explored the cognitive and psychosocial benefits of serious gaming for persons with dementia, minimal research has explored satisfaction with game‐like technologies for dementia education among older adults in low‐income housing communities. This preliminary study aimed to explore levels of satisfaction with a Kahoot Dementia Trivia, which was designed to foster awareness and understanding of dementia and its subtypes, amongst older adults residing in a subsidized housing community (Aim 1). We aimed to identify health‐related topics of interest for future Kahoot Trivia (Aim 2).

**Method:**

Ten older adults who resided in a senior subsidized housing community in Greenville, South Carolina were recruited. Participants engaged in a Kahoot Dementia Trivia activity and completed a Likert‐scale satisfaction survey assessing enjoyment, social engagement, future gameplay, recommendation for game play for other individuals (Aim 1). Higher rating was indicative of positive responses. Additionally, participants were provided 11 topics and were asked to choose the topics of greatest interest for future Kahoot Trivia games (Aim 2). Frequencies and descriptives were calculated.

**Result:**

On average, participants noted very high levels of enjoyment (M=9.30; SD=1.64; range=5‐10), felt the Kahoot Dementia Trivia encouraged social engagement (M=9.20; SD=2.20; range=3‐10), with very high likelihood of participating in this activity in the future (M=9.10; SD=2.51; range=2‐10; Aim 1). On a scale of 1‐5, 70% were likely/very likely (*n* = 7) to encourage others to attend this type of activity (Aim 1). Of the 11 potential Kahoot Trivia topics of interest, participants reported the following: 60% (*n* = 6) selected dementia, 60% (*n* = 6) selected mindfulness, 50% (*n* = 5) selected Alzheimer's disease, and 50% (*n* = 5) selected diabetes (Aim 2).

**Conclusion:**

Dementia education as facilitated through technological game‐like experiences demonstrated overall high levels of satisfaction, social engagement, and future participation interest with participants demonstrating high likelihood for recommending this and similar activities to others. Interestingly, mindfulness‐based education, as offered through Kahoot Trivia may be indicative of alternative approaches to stress coping and management, which warrants additional exploration.

